# Uncoupling GP1 and GP2 expression in the Lassa virus glycoprotein complex: implications for GP1 ectodomain shedding

**DOI:** 10.1186/1743-422X-5-161

**Published:** 2008-12-23

**Authors:** Megan M Illick, Luis M Branco, Joseph N Fair, Kerry A Illick, Alex Matschiner, Randal Schoepp, Robert F Garry, Mary C Guttieri

**Affiliations:** 1 BioFactura, Inc., Rockville, MD, USA; 2 Tulane University Health Sciences Center, New Orleans, LA, USA; 3 Tulane University School of Public Health & Tropical Medicine, New Orleans, LA, USA; 4 Virology Division, United States Army Medical Research Institute of Infectious Diseases, Fort Detrick, MD, USA; 5 Department of Science, Cedar Crest College, Allentown, PA, USA; 6 Diagnostic Systems Division, United States Army Medical Research Institute of Infectious Diseases, Fort Detrick, MD, USA

## Abstract

**Background:**

Sera from convalescent Lassa fever patients often contains antibodies to Lassa virus (LASV) glycoprotein 1 (GP1), and glycoprotein 2 (GP2); Immunization of non-human primates with viral vectors expressing the arenaviral glycoprotein complex (GPC) confers full protective immunity against a lethal challenge with LASV. Thus, the development of native or quasi native recombinant LASV GP1 and GP2 as soluble, uncoupled proteins will improve current diagnostics, treatment, and prevention of Lassa fever. To this end, mammalian expression systems were engineered for production and purification of secreted forms of soluble LASV GP1 and GP2 proteins.

**Results:**

Determinants for mammalian cell expression of secreted uncoupled Lassa virus (LASV) glycoprotein 1 (GP1) and glycoprotein 2 (GP2) were established. Soluble GP1 was generated using either the native glycoprotein precursor (GPC) signal peptide (SP) or human IgG signal sequences (s.s.). GP2 was secreted from cells only when (1) the transmembrane (TM) domain was deleted, the intracellular domain (IC) was fused to the ectodomain, and the gene was co-expressed with a complete GP1 gene in *cis*; (2) the TM and IC domains were deleted and GP1 was co-expressed in *cis*; (3) expression of GP1 was driven by the native GPC SP. These data implicate GP1 as a chaperone for processing and shuttling GP2 to the cell surface. The soluble forms of GP1 and GP2 generated through these studies were secreted as homogeneously glycosylated proteins that contained high mannose glycans. Furthermore, observation of GP1 ectodomain shedding from cells expressing wild type LASV GPC represents a novel aspect of arenaviral glycoprotein expression.

**Conclusion:**

These results implicate GP1 as a chaperone for the correct processing and shuttling of GP2 to the cell surface, and suggest that native GPC SP plays a role in this process. In the absence of GP1 and GPC SP the GP2 protein may be processed by an alternate pathway that produces heterogeneously glycosylated protein, or the polypeptide may not fully mature in the secretory cascade in mammalian cells. The expression constructs developed in these studies resulted in the generation and purification of soluble, uncoupled GP1 and GP2 proteins from mammalian cells with quasi-native properties. The observation of GP1 ectodomain shedding from cells expressing wild type LASV GPC establishes new correlates of disease progression and highlights potential opportunities for development of diagnostics targeting the early stages of Lassa fever.

## Background

LASV, a member of the *Arenaviridae *family, causes a severe, often fatal, hemorrhagic fever that is endemic to West Africa; where as many as 300,000–500,000 infections occur per year [1-3]. The case fatality rate for hospitalized Lassa fever patients is 15%–20%, and during epidemics, the rate can reach as high as 50% [4,5]. The virus is maintained in nature by its peridomestic rodent host, *Mastomys natalensis*, and is primarily transmitted to humans by aerosolized urine of infected animals, though severe nosocomial outbreaks and imported cases of Lassa fever in non-endemic areas have been documented [5,6]. Presently, there is no licensed vaccine or anti-viral therapy available for the prevention or treatment of this disease, and there is no commercially available Lassa fever diagnostic assay. The threat posed by LASV is heightened further by the potential use of the virus as a biological weapon, which is substantiated by the stability of the virion, demonstrated person-to-person transmission, the severity of disease, lack of therapeutic and prophylactic reagents, and the capacity for aerosolization. As a result, LASV is classified as a Category A Priority Pathogen and biosafety level (BSL)-4 agent by the Centers for Disease Control and Prevention. Collectively, these factors underscore the need for effective diagnostics, vaccines, and therapies against Lassa fever. In this regard, production and characterization of native or quasi-native recombinant LASV glycoproteins would facilitate efforts to generate effective countermeasures.

The LASV genome is comprised of two ambisense, single-stranded RNA molecules, designated large (L) and small (S), which are contained in a nucleoprotein capsid encompassed by an outer envelope displaying surface glycoprotein spikes [7]. The L segment encodes the viral polymerase (L protein) and RING finger Z matrix protein; whereas, the S segment encodes the glycoprotein precursor (GPC), which is 76-kDa in length, and a 63-kDa nucleoprotein (NP). Cleavage of GPC by the protease SKI-1/S1P at the recognition motif RRLL results in the N-terminal 42-kDa glycoprotein 1 (GP1) subunit and the C-terminal 38-kDa glycoprotein 2 (GP2) subunit containing a transmembrane (TM) and intracellular (IC) domain [8]. GP1 mediates virus binding to the cellular glycoprotein receptor alpha-dystroglycan while the structure of GP2 is consistent with viral TM fusion proteins [9,10]. GPC contains a 58 residue hydrophobic N-terminal signal peptide (SP), which directs the precursor to the endoplasmic reticulum (ER) for further processing [11]. The SP, which has been implicated in membrane fusion, may also serve a role in proteolytic processing of the glycoprotein and, as suggested for Junin virus (JUNV), assembly of the glycoprotein complex [12,13].

Understanding the elaborate and complex interactions between the SP, SKI-1/S1P proteolytic pathway, and the glycoprotein complex would facilitate the generation of prophylactic and therapeutic strategies. To this end, an array of plasmids was engineered that permitted optimal mammalian-based expression of the LASV glycoproteins, including GPC, GP1, and GP2. Various signal peptides, purification tags, and modifications to internal domains were employed for the generation and characterization of soluble, uncoupled, full-length, quasi-native LASV GP1 and GP2. Parameters required for efficient expression were determined, affording valuable insight into LASV glycoprotein processing and identifying glycoprotein variants that may have significant implications in the pathogenesis of LASV in humans.

## Results

### Expression and purification of soluble LASV GP1

**Figure 1:**
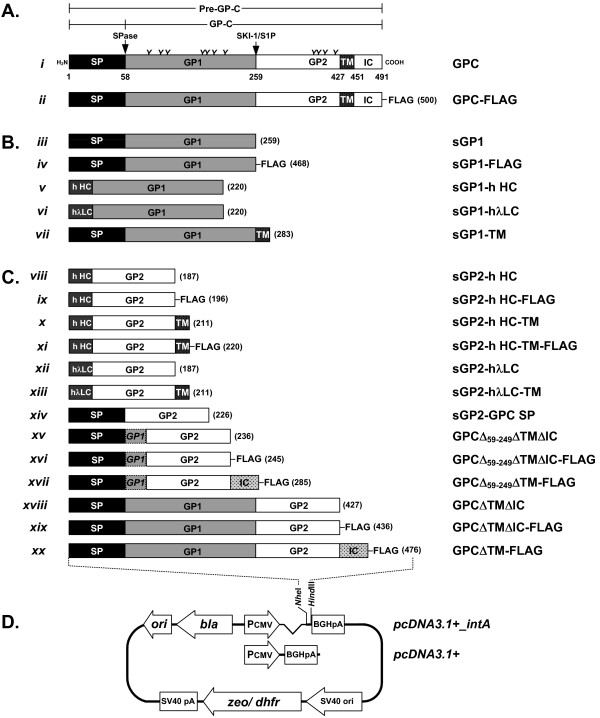
**Graphic representation of LASV GPC, GP1, and GP2 constructs employed in the expression of glycoproteins in mammalian cells**. The LASV Josiah GPC gene was the backbone for all glycoprotein expression constructs. (A. *i*) The 58 amino-acid signal peptide (SP) which is post-translationally cleaved by SPase precedes the 201 amino acid GP1 ORF (59 – 259). The GP2 ORF spans amino acids 260 – 491. The GP2 gene contains an ectodomain of 168 amino acids, followed by a 24 amino acid transmembrane (TM) domain, and a 40 amino acid intracellular (IC) domain. The cleavage positions of SPase and SKI-1/S1P proteases are indicated by arrows. The relative position of 7 N-linked glycosylation sites in GP1 and 4 in GP2 are indicated by ***Y ***symbols. Constructs for expression of GPC-FLAG (A. *ii*), GP1 (B), GP2 and soluble GPC (C), are noted. The pcDNA3.1(+) plasmid background was used for expression of glycoprotein constructs (D).

**Figure 2:**
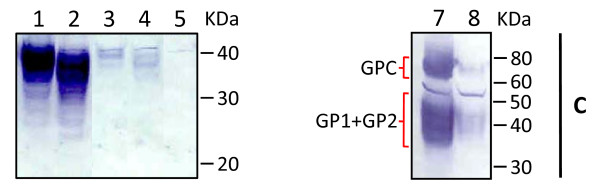
**Intracellular expression of LASV GP1TM, sGP1, and wild type GPC from mammalian vectors driven by CMV MIE intron-A containing or intronless constructs**. Ten micrograms of total protein from HEK-293T/17 cell extracts (C) transfected with each DNA construct were resolved on SDS-PAGE gels, blotted onto nitrocellulose membranes and probed with a mix of LASV GP1-specific mAbs and an HRP-conjugated goat α-mouse IgG antibody. Protein expression from CMV intron-A containing GP1-TM (lane 1) and sGP1 (lane 2) constructs were compared to intronless counterparts (lane 3, GP1-TM; lane 4, sGP1). An empty plasmid control, pcDNA3.1(+):intA, is shown in lane 5. Wild type GPC expressed from an intron-A containing construct (lane 7) was compared to an intronless counterpart (lane 8). Expression of GPC was detected with a mix of LASV GP1 and GP2-specific mAbs and an HRP-conjugated goat α-mouse IgG antibody. Protein molecular weight sizes in kDa are indicated to the right of the panel.

Constructs expressing the native GPC SP through the C-terminal end of mature GP1 or the same configuration fused at the C-terminus to the TM domain of mature GP2 were engineered for production of soluble GP1 (sGP1) or membrane-anchored GP1 (GP1-TM), respectively (Figure 1B iii and vii). Expression was achieved at high levels in HEK-293T/17 cells using the cytomegalovirus (CMV) major immediate early (MIE) promoter containing the intron-A sequence. When compared to intronless counterparts, the intron-A sequence greatly enhanced intracellular expression of GP1-TM and sGP1 (Figure 2, lanes 1–4). Expression of wild type GPC was similarly enhanced by CMV intron-A (Figure 2, lanes 7 and 8). The multiple banding patterns observed in GPC and GP1 expression profiles likely reflected differences in glycosylation at the time of sample preparation. High-level expression of sGP1 was achieved irrespective of the signal sequence fused to the N-terminus of the gene. The native GPC SP, a 19 amino acid (a.a.) human IgG light-chain (hλLC) signal sequence (s.s.), or a 19 a.a. human IgG heavy-chain (hHC) s.s. all resulted in the secretion of full length and homogeneously glycosylated sGP1 and FLAG-tagged sGP1 (sGP1-FLAG) (data not shown). As a result of these data, subsequent studies, including co-transfections with GP2-expressing constructs, were performed with GPC SP-driven sGP1 or sGP1-FLAG using the CMV MIE promoter containing intron-A.

**Figure 3:**
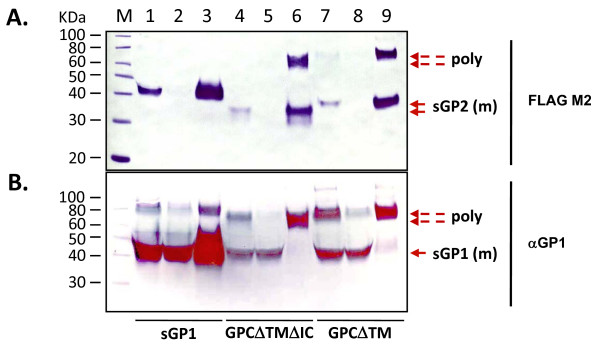
**Purification of sGP1-FLAG, GPCΔTMΔIC-FLAG, and GPCΔTM-FLAG from transiently transfected HEK-293T/17 cell supernatants**. Supernatants from 72-hour transient transfections were subjected to IP using an ANTI-FLAG M2 agarose gel. Twenty microliters of cleared supernatant (lane 1), unbound fraction (lane 2), and eluate fraction (lanes 3) were resolved on 10% SDS-PAGE gels, blotted onto nitrocellulose membranes and probed with anti-FLAG M2 mAb and a goat α-mouse IgG-HRP (panel A). The blot was stripped and reprobed with anti-GP1 mAb 2074 and the same secondary as above (panel B). The monomer sGP1 and sGP2 species (m) on each blot are indicated by solid arrows. The GP1+GP2 polyprotein on each blot (poly) are indicated by broken arrows. MagicMark XP western blot molecular weight markers (M), with sizes (kDa) are shown to the left of the panel. The construct designations for each transfection supernatant are indicated below the figure. In both panels lanes 1, 4, and 7 correspond to supernatant load; lanes 2, 5, and 8 are unbound supernatant fractions; lanes 3, 6, and 9 are eluate fractions.

**Figure 4:**
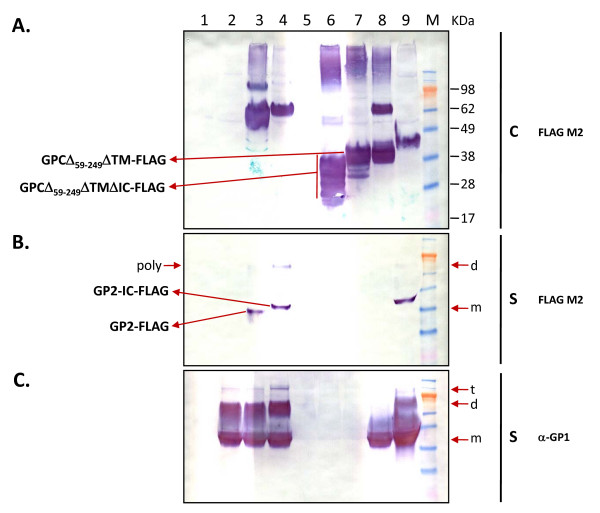
**Intracellular and secreted forms of LASV GP2 variants and co-transfections with sGP1 in HEK-293T/17 cells**. Cell extracts and supernatants from HEK-293T/17 transfected with LASV GP2 variants or from co-transfections with sGP1 were analyzed for the expression and secretion of GP1 and GP2. Ten micrograms of transfected cell protein were resolved on SDS-PAGE gels, blotted onto nitrocellulose membranes and probed with anti-LASV GP2 (panel A) or GP1 mAbs (panel B). Similarly, twenty μL of supernatant from each transfection were resolved on SDS-PAGE gels, blotted, and probed with the same anti-GP2 (panel C) and anti-GP1 mAbs (panel D). Control plasmid pcDNA3.1(+):intA (lane 1) was transfected alongside constructs sGP2-h HC (lanes 2), sGP2-hλLC (lanes 3), and sGP2-GPC SP (lane 4). The sGP2 constructs were co-transfected in the same order in lanes 5, 6, and 7 with equimolar amounts sGP1 expression plasmid. A transfection with sGP1 construct is shown in lane 8. Molecular weight markers with sizes (kDa) are shown to the left of the panel. Panels showing expression profile from cell extracts (C) and supernatants (S) are demarcated by vertical lines, along with the respective anti-LASV mAb probes. The co-transfection profiles with GP1 and GP2 constructs are indicated by (-) and (+) symbols at the bottom of the figure.

The predominant sGP1-FLAG species detected was a homogeneous 42-kDa monomeric form of the protein, which was obtained from cells transfected with vector sGP1-FLAG (Figure 3B, lane 3). A species of ca. 85-kDa, which was only detected by a GP1-specific monoclonal antibody (anti-GP1 mAb 2074), corresponded to a homodimer of sGP1-FLAG, which was not easily disrupted by denaturing and reducing conditions (Figure 3B, lane 3; Figure 4C, lanes 2–4 and lane 9). A minor trimerized form of sGP1-FLAG was also detected (Figure 4C, lanes 2–4 and lane 9).

Our studies indicated that approximately 50% of the sGP1-FLAG protein could not be captured by FLAG resin affinity chromatography. This result was not related to insufficient binding capacity of the resin and was evident only when comparing data from Western blot analysis using anti-GP1 mAb 2074 to results obtained from similar analyses using antibody specific for FLAG (FLAG M2 mAb) (Figure 3A and 3B, lane 2). Unbound sGP1-FLAG could not be captured by reprocessing through fresh resin (data not shown). The FLAG tag was readily detected by Western blot in the eluted GP1 protein fraction using both anti-GP1 and FLAG M2 mAbs (Figure 3A and 3B, lane 3). Collectively, these results suggested that sGP1-FLAG was partially cleaved by SKI-1/S1P protease, as the enzyme's recognition sequence was retained in the construct and was directly fused to FLAG (RRLL↓DYKDDDDKG).

### Expression of soluble and membrane-anchored GP2

As determined for sGP1, expression of sGP2 was significantly higher when driven by a CMV promoter containing intron-A (data not shown), resulting in the selection of this promoter for all GP2 analyses. Unlike GP1, neither native GPC SP nor light- or heavy-chain IgG s.s. elicited effective expression, processing, and secretion of GP2 when fused to the ectodomain of the protein (Figure 4A and 4C, lanes 2–4). Irrespective of the s.s. employed, intracellular GP2 expression resulted in highly heterogeneously glycosylated species of the protein (Figure 4A, lanes 2–4). Inclusion of the GP2 TM domain in constructs GP2 hHC-TM and GP2-λLC-TM, as outlined in Figure 1C x and xiii, respectively, resulted in undetectable levels of GP2 (data not shown). Thus, expression of membrane-anchored GP2 was not pursued in subsequent studies.

### Co-expression of GP1 and GP2 in *trans*

**Figure 5:**
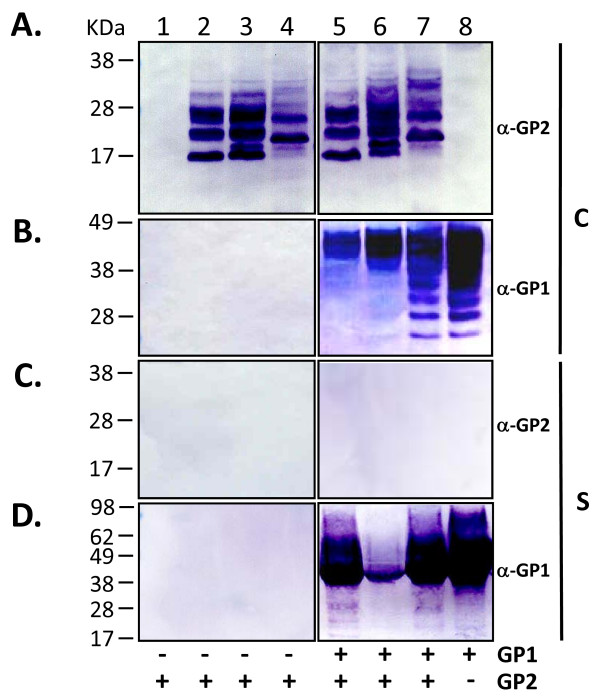
**Expression and secretion of sGP2 from LASV GPC deletion variants**. Cell extracts and supernatants from HEK-293T/17 transfected with LASV GPC deletion variants were analyzed for the expression and secretion of GP1 and GP2 proteins. Ten micrograms of total protein from transfected cells were resolved on SDS-PAGE gels, blotted onto nitrocellulose membranes and probed with FLAG M2 mAb (A). Twenty μL of supernatant from the corresponding samples were similarly resolved, blotted, and probed with FLAG M2 mAb (B) or anti-LASV GP1 mAbs (C). (Lane 1) control plasmid pcDNA3.1(+):intA, (lane 2) GPCΔTMΔIC, (lane 3) GPCΔTMΔIC-FLAG, (lane 4) GPCΔTM-FLAG, (lane 5) GPCΔ_59–249 _ΔTMΔIC, (lane 6) GPCΔ_59–249_ΔTMΔIC-FLAG, (lane 7) GPCΔ_59–249_ΔTM-FLAG, (lane 8) GPC-FLAG, (lane 9) sGP1-FLAG. SeeBlue^® ^Plus2 pre-stained molecular weight markers (M), with sizes (kDa) are shown to the right of the panel. Designations for the constructs that generated the intracellular expression pattern in lanes 6A and 7A are displayed to the left of panel A. Similarly, designations that generated the secreted expression pattern in lanes 3B and 4B are displayed to the left of panel B. Positions of monomer (m), dimer (d), and trimer (t) forms of the GP1 protein are indicated by arrows. Similarly, the position of monomeric (m) and dimerized (d) forms of sGP1 (right), and polyprotein (poly) species consisting of sGP1+sGP2 (left) in panel B are indicated by arrows.

HEK-293T/17 cells were co-transfected with various sGP2 constructs (Figure 1C viii, xii, xiv) and expression vector sGP1 (Figure 1B iii) to determine the co-translational processing and secretion efficiency of the glycoprotein complex when expressed in *trans*. All GP2 constructs exhibited similar intracellular expression patterns, including heterogeneous glycosylation, irrespective of the presence of GP1 (Figure 4A, lanes 2–7). GP2 constructs containing a human IgG s.s. co-transfected with construct sGP1 consistently resulted in more homogeneously glycosylated intracellular GP1 (Figure 4B, lanes 5–8). None of the transfection formats resulted in detectable levels of sGP2 in cell culture supernatants (Figure 4C, lanes 2–7); whereas, expression of sGP1 was confirmed in both cell extracts and supernatants (Figure 5B and 5D, lanes 5–8), though diminished levels of sGP1 were detected in the supernatant of cells co-transfected with sGP2-λLC (Figure 4D, lane 6).

### Co-expression of GP1 and GP2 in *cis*

To identify regions of GPC required for secretion of homogenous GP1 and GP2, several constructs were engineered with or without the TM or IC domains, as depicted in Figure 1. Using the FLAG M2 mAb, GP2 was not detected in extracts prepared from cells transfected with either GPCΔTMΔIC-FLAG or GPCΔTM-FLAG (Figure 5A, lanes 3 and 4); however, the protein was observed in cell culture supernatants (Figure 5B, lanes 3 and 4). Conversely, these constructs yielded GP1 in cell extracts (data not shown) and supernatants (Figure 5C, lanes 3 and 4), with detection performed using anti-GP1 mAb 2074. Furthermore, cells transfected with GPCΔTMΔIC, GPCΔTMΔIC-FLAG, GPCΔTM-FLAG, and sGP1-FLAG produced significant levels of sGP1 dimers and trimers (Figure 5C, lanes 2–4 and lane 9). Transfection with GPCΔTM-FLAG, which contained the IC domain, generally produced more secreted GP2 per relative unit volume, but the domain was not required for generation of fully glycosylated and homogeneous protein (Figure 5B, lanes 3 and 4). Results similar to those described above were obtained when detection was performed using a GP2-specific mAb (data not shown).

GP2 was detected in extracts but not supernatants of cells transfected with constructs containing only the C-terminal residues that code for the SKI-1/S1P protease recognition site (Figure 5A and 5B, lanes 6 and 7). This pattern of GP2 cellular retention was also observed with constructs sGP2-h HC, sGP-hλLC, and sGP2-GPC SP, all of which did not contain the GP1 ORF (Figure 4, lanes 2–4). GP2 with highly heterogeneous size distribution, indicating different levels of glycosylation, was generated when the IC domain was omitted in addition to deletion of most of the GP1 open reading frame (ORF) (figure 5A, lane 6), and as expected, sGP1 was not detected in this format (Figure 5C, lanes 5–7). Conversely, intracellular expression primarily yielded fully glycosylated GP2 of approximately 38-kDa in size when the IC domain was present (Figure 5A, lane 7). GPC variants from constructs lacking the IC domain resulted in significant levels of high molecular weight aggregates, particularly when GP1 was not co-expressed (Figure 5A, lanes 3, 6 and 7). This pattern was not observed in cells transfected with construct GPCΔTM-FLAG, which also yielded the highest levels of secreted GP2 (Figure 5A, lane 4), nor was it evident in cells transfected with sGP1 (Figure 5A, lane 9). High molecular weight aggregates were also evident in cells expressing wild type GPC, although at lesser levels than with constructs GPCΔ_59–249_ΔTMΔIC-FLAG and GPCΔ_59–249_ΔTM-FLAG (Figure 5A, lane 8).

### Glycosylation of sGP1 and sGP2

**Figure 6:**
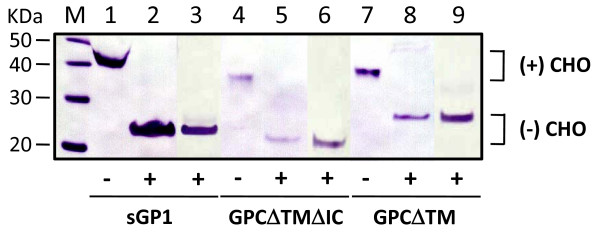
**PNGase F and Endo H treatment of sGP1-FLAG, GPCΔTMΔIC-FLAG, and GPCΔTM-FLAG from transfected cell supernatants**. Twenty μL of each FLAG M2 resin-purified protein from a typical transfection, as shown in figure 3, were subjected to treatment with 500 U of PNGase F or Endo H, as described in materials and methods. The reactions were resolved on SDS-PAGE gels alongside untreated counterparts, blotted, and probed with anti-FLAG M2 mAb and an HRP-labeled goat α-mouse IgG. Soluble GP1-FLAG (lanes 1, 2, 3), GPCΔTMΔIC-FLAG (lanes 4, 5, 6), and GPCΔTM-FLAG (lanes 7, 8, 9) show different mobilities based on whether proteins were treated with PNGase F (lanes 2, 5, 8), Endo H (lanes 3, 6, 9), or were left untreated (lanes 1, 4, 7). MagicMark XP™ protein molecular weight markers (M), with sizes (kDa) are shown to the left of the panel. Glycosylated species are denoted by (+) CHO, and deglycosylated counterparts by (-) CHO. The addition of amydase to each reaction is denoted by (+) symbol below the figure, whereas untreated controls are marked by (-).

To examine glycosylation patterns, sGP1 and sGP2 produced in sGP1-FLAG, GPCΔTMΔIC-FLAG, or GPCΔTM-FLAG transfected cells were subjected to cleavage with (1) PNGase F, an amidase that cleaves between the innermost GlnNAc and asparagines residues of high mannose, hybrid, and complex oligosaccharides from N-linked glycoproteins [14]; or (2) Endo H, a glycosidase that cleaves the chitobiose core of high mannose and some hybrid oligosaccharides from N-linked glycoproteins [14]. Based on protein molecular weights following cleavage with PNGase F or Endo H, sGP1 and sGP2 were secreted as glycosylated proteins, with noted reductions from 42-kDa to 23-kDa for sGP1-FLAG (Figure 6, lanes 1–3), 36-kDa to 21-kDa for GPCΔTMΔIC-FLAG (Figure 6, lanes 4–6), and 38-kDa to 24-kDa for GPCΔTM-FLAG (Figure 6, lanes 7–9).

### Expression profiles of sGP1 and sGP2 from GPC variants

Western blot analysis using the anti-FLAG M2 mAb detected a single, homogenous 36-kDa sGP2 species in the supernatant of cells transfected with GPCΔTMΔIC-FLAG (Figure 3A, lane 4). Following FLAG M2 gel affinity purification, this protein was captured by the resin, as indicated by the lack of trace detection in the unbound fraction (Figure 3A, lane 5). Analysis of the eluate fraction revealed two strong protein bands, a 36-kDa sGP2 monomer and a larger species ca. 72-kDa in size (Figure 3A, lane 6). Supernatant prepared from cells transfected with GPCΔTM-FLAG revealed a 38-kDa sGP2 species (Figure 3B, lane 7), and in the eluate fraction, two strong proteins were detected, a 38-kDa sGP2 monomer and a larger species ca. 76-kDa in size (Figure 3B, lane 9). As GPCΔTMΔIC-FLAG and GPCΔTM-FLAG constructs generate full length GP1 and truncated sGP2 variants, the blots were subsequently stripped and reprobed with anti-GP1 mAb 2074 to confirm the presence and expression level of GP1 in purified preparations. Western blot analyses of the supernatant load of each GPC variant detected two bands, a 42-kDa sGP1 monomer and a 78-kDa species (Figure 3B, lanes 4 and 7). The monomeric form of sGP1 was also readily detected in the unbound fraction (Figure 3B, lanes 5 and 8). The eluates from both GPC variants revealed very strong bands ca. 76-kDa and 78-kDa in size, for GPCΔTMΔIC-FLAG and GPCΔTM-FLAG, respectively (Figure 3B, lanes 6 and 9). These proteins co-localized on the gel with proteins detected using anti-FLAG M2 mAb (Figure 3A, lanes 6 and 9). Collectively, these data suggested that sGP1 and sGP2 form stable, covalently-bonded heterodimer complexes or that a significant fraction of the GPCΔTMΔIC-FLAG- and GPCΔTM-FLAG-generated proteins are uncleaved intracellularly by SKI-1/S1P protease and are thereby secreted in polyprotein form. Anti-GP1 mAb 2074 did not detect significant monomeric sGP1 in the eluate fractions, indicating that the protein is associated with sGP2 (Figure 3B, lanes 6 and 9). A very minor component ca. 110-kDa in size was detected in most preparations of GPCΔTM-FLAG using anti-GP1 mAb 2074, which potentially represented additional multimeric complexes (Figure 3B, lanes 7 and 9). These results indicated that approximately 50% of the sGP2 generated from GPCΔTMΔIC-FLAG and GPCΔTM-FLAG is associated with sGP1, whereas ~50% is monomeric protein (Figure 3A, lanes 6 and 9).

## Discussion

Production of uncoupled glycosylated, quasi-native LASV GP1 and GP2 proteins was achieved by engineering constructs comprised of mammalian cell-specific expression elements and variants of the GPC complex. The addition of CMV intron-A was necessary to elicit highly efficient expression of all GPC variants, reflecting the importance of this element in the post-transcriptional processing of the viral messenger RNA. Three SP were successfully used for expression and translocation of sGP1, including native GPC SP as well as human IgG λ light-chain and heavy-chain s.s. The two IgG-derived SP were selected as they are very efficient secretory signals and fusion of these domains to heterologous recombinant proteins have been shown to enhance expression and secretion from mammalian cells [15], (Branco, Guttieri, Garry, unpublished data). Previous studies examining SP substitutions and GPC expression have not met with similar success. Substitution of the native GPC SP with CD8 α-chain or Influenza virus hemagglutinin (HA) s.s. permitted translocation of GPC to the ER; however, the protein was not further proteolytically processed by SKI-1/S1P into GP1 and GP2 (Eichler *et al*., 2003). Likewise, Agnihothram *et al*. (2006) demonstrated that the JUNV GPC SP was required for protein transport to the Golgi and export to the cell surface. In addition, the cytoplasmic domain of GP2 encodes a dibasic a.a. motif that is widely utilized in the retrieval of TM proteins to the ER and that binding of the SP to this motif masks localization to this organelle, thereby facilitating the mobilization of the glycoprotein complex for transit through the Golgi [13]. Although the GPC SP and IgG-derived s.s. were effective for sGP1 expression, all versions of uncoupled sGP2 containing the same signal sequences were not effectively processed, yielding heterogeneously glycosylated species that were detected only in cell extracts. The relative levels of expression and pattern of heterogeneous glycosylation were similar with each of the s.s. employed, thereby implicating additional requirements for full post-translational processing of this protein. Based on these results and published observations, the native GPC SP was selected for the majority of the studies reported here.

To elucidate the elements critical for sGP2 secretion, various GP1- and GP2-expressing constructs were co-expressed in *trans *and *cis*, thereby providing a means to identify a role, if any, for GP1 as a chaperone or co-factor in the processing and trafficking of GP2. In addition, the SKI-1/S1P protease cleavage site was investigated as a possible determinant for correct processing of GP2, and the previously identified dibasic amino acid retention signal in the IC domain of GP2 was evaluated as a modulator of protein processing and exporting for sGP2 variants.

Co-transfection of vector sGP1 with all three variants of sGP2 containing only the ectodomain of the protein did not result in expression and secretion of detectable levels of GP2. In each instance, similar levels of intracellular GP2 expression were observed, albeit resulting in heterogeneous glycosylation. Higher levels of homogeneously glycosylated GP1 were observed in extracts from cells co-transfected with sGP1 and GP2-expressing constructs containing IgG-derived SP. Furthermore, the levels of secreted GP1 from cells co-transfected with sGP1 and sGP2-hλLC were consistently lower than with sGP2-hHC, sGP2-GPC SP, or when compared with sGP1 alone. Although the mechanisms contributing to these observations were not determined by these studies, the implications of different s.s. in the processing efficiency of LASV glycoproteins were evident. These results clearly demonstrated that expression of GP1 in *trans *cannot influence the co-expression, correct post-translational processing, and secretion of GP2 from 293T cells.

In contrast, when the ectodomain of GP2 was expressed with full length GP1 in *cis *and was driven by the GPC SP, the resulting sGP2 was very efficiently processed and secreted from cells (Figure 5B, lanes 3 and 4). Furthermore, the protein was homogeneously glycosylated (Figure 5B, lanes 3 and 4). Constructs containing a truncated GP1 that encoded only the C-terminal 10 amino acids of the ORF, which included the cleavage domain recognized by SKI-1/S1P protease in addition to the ectodomain of GP2, did not result in detectable secretion of GP2 protein. Moreover, the inclusion of the GP2 IC domain did not influence the correct processing of GP2 unless a full length GP1 ORF was co-expressed in *cis*. When only the GPC SP, Δ_59–249_, and the GP2 ectodomain were present, either with or without the IC domain, the resulting intracellular GP2 was consistently heterogeneously glycosylated. However, in repeated studies the presence of a GP2 IC domain resulted in less heterogeneously glycosylated GP2, further implicating the role of the previously identified ER retention signal in the correct processing of the GPC complex. Without *cis *expression of GP1, the inclusion of the GP2 IC resulted only in a semi-homogeneously glycosylated GP2 intermediate species that could not be secreted, providing rationale for involvement of full length GP1 in the final stage of the export of the SP-GP1-GP2 complex to the cell surface. Although GPCΔTMΔIC-FLAG and GPCΔTM-FLAG both resulted in the secretion of homogeneously glycosylated sGP2, the inclusion of the IC domain resulted in higher volumetric protein production levels, perhaps due to more efficient processing of the chimeric complex via the association of the SP with the protein and efficient mobilization through the Golgi. The co-expression of a full length GP1 with GP2 in *cis*, driven by the GPC SP and containing the GP2 IC domain, comprised the shortest module for efficient processing and secretion of homogeneously glycosylated GP2.

These studies also revealed that expression of the GPC complex often results in significant aggregation of glycoprotein components in the intracellular environment. Insoluble aggregates greater than 98-kDa, which were not dispersed by denaturing and reducing agents (*e.g*., SDS and DTT), were readily detected. These aggregates were evident in transfections using the wild type GPC construct and, more so, using GPCΔ_59–249_ΔTMΔIC-FLAG and GPCΔ_59–249_ΔTM-FLAG. Construct GPCΔTMΔIC-FLAG generated less aggregate than its Δ_59–249 _counterpart, with a pronounced species at ca. 100-kDa that corresponded to twice the size of an unprocessed molecule. In addition, less detectable aggregate was generated with GPCΔTM-FLAG. Furthermore, visible absence of GP2 in extracts from cells transfected with GPCΔTMΔIC-FLAG and GPCΔTM-FLAG constructs pointed to very rapid and efficient processing and secretion of sGP2 into the extracellular environment, in stark contrast to that observed with Δ_59–249 _counterparts and, to some extent, sGP1-FLAG. Whether the apparent sGP2 secretion kinetics played a role in the formation of less intracellular aggregate is speculative at this juncture. However, these observations highlighted differences in secretion kinetics for individual LASV glycoproteins. Whereas GP2 can be efficiently and nearly completed exported from the cell when all co-factors are present, GP1 displays slower temporal secretory kinetics, with significant levels of the protein being detected intracellularly at any given point during the transient transfection timeline. It can be reasoned that the newly identified role of co-factor or chaperone for GP1 is in-line with its slower secretory kinetics, as it would ensure more efficient processing of GP2, which cannot be correctly processed and exported on its own. As these studies point to the crucial role of GP1 expression in *cis *with GP2 for efficient processing and secretion of the latter, both proteins can be readily detected in the extracellular environment of cells transiently transfected with GPC variants (Figure 5B, C, lanes 3, 4). Thus, a highly selective purification process will be required to ensure that preparations of sGP2 are free of GP1 protein contaminants.

It has been proposed that GP1 associates non-covalently with GP2 on the surface of cells [16-18]. This interaction should be susceptible to disruption by treatment with anionic detergents commonly used in PAGE buffers. However, our Western blot detection of a prominent high molecular component purified from the supernatants of cells transfected with GPCΔTMΔIC-FLAG and GPCΔTM-FLAG constructs suggested that either sGP1 and sGP2 formed a highly stable heterodimer complex or that uncleaved GPC polyprotein variants were secreted. If a heterodimer was generated, it was not disrupted by denaturing and reducing conditions, which suggested that a unique covalent association existed between the two proteins. Alternatively, secreted forms of each unprocessed polyprotein could have resulted from presence of the SP and the absence of a TM domain. These polyproteins appeared fully glycosylated, as their size of ca. 76-kDa corresponded to combined sizes of GP1 (ca. 42-kDa) and GP2 (ca. 36 – 38-kDa) containing a full complement of carbohydrate. Unglycosylated counterparts migrating in the 42-kDa range were not detected.

In transient expression experiments, the production profile of wild type GPC and variants should reveal full length uncleaved complex, as well as fully processed GP1 and GP2 subunits, with the time of harvest representing a snapshot of cellular events. Expression of wild type GPC in 293T/17 cells consistently resulted in the detection of a ca. 76-kDa species corresponding to GPC, as well as processed GP1 and GP2 subunits (42-kDa and 38-kDa, respectively). Conversely, GPC gene expression from GPCΔTMΔIC-FLAG and GPCΔTM-FLAG constructs primarily yielded ca. 68-kDa and 70-kDa species, respectively, in the intracellular protein fraction. Processed, monomeric GP1 from these constructs was readily detected with anti-GP1 mAbs (data not shown), but GP2-FLAG was only detected in the corresponding cell supernatants. Probing these blots with anti-GP2 mAbs resulted in the same lack of detection of GP2 as with anti-FLAG M2 reagent, thus confirming the absence of significant levels of GP2 intracellularly. As outlined above, such observations point to differential processing and secretion of GP1 and GP2 from both GPC variants, supporting the view that GP2 was rapidly secreted from the intracellular environment and that GP1 was processed via slower secretory kinetics.

It is also unclear at this juncture whether GPCΔTMΔIC-FLAG and GPCΔTM-FLAG proteins are specifically but inefficiently cleaved by SKI-1/S1P protease as a result of normal virus protein processing or if this phenomenon is related to the elevated expression levels from the very strong CMV promoter and associated intron-A sequence employed in these studies. It is possible that high level expression of arenaviral GPC overwhelms post-translational cellular processing mechanisms, thus resulting in a large fraction of uncleaved protein.

Future studies aimed at finely dissecting the steps involved in arenaviral GPC processing could benefit from the use of weaker promoters, such as the basal tyrosine kinase (TK) elements commonly used to drive expression of selectable markers in eukaryotic cells [19,20]. Although the kinetics of GPC processing by SKI-1/S1P protease are not well understood, a protein concentration-dependent steady state mechanism could be easily overwhelmed by the significantly elevated levels of polypeptide synthesis mediated by CMV promoters in eukaryotic cells. Thus, these results cannot be used as a correlate for wild type GPC protein processing efficiency in LASV infected cells.

The strong sGP1-FLAG band detected with anti-GP1 mAb but not with anti-FLAG mAb in the unbound fraction following capture of the protein with FLAG M2 gel resin points to the existence of two different variants of this protein. The sGP1-FLAG used in these studies contained the native recognition site for SKI-1/S1P protease fused to the FLAG domain. It is likely that a significant fraction of the sGP1-FLAG protein was cleaved by SKI-1/S1P protease, thus generating untagged and FLAG-tagged variants. Where it concerns the future development of purification schemes, it may be possible to purify both versions of sGP1 from the same supernatant pool: sGP1-FLAG can be captured by FLAG-M2 resin and the flow through, containing untagged protein, could subsequently be purified by LASV GP1 affinity chromatography. Alternatively, mutating the SKI-1/S1P protease recognition domain from RRLL to RRAA would abrogate cleavage of the downstream purification tag and thus increase yields of recombinant protein via a single chromatography step (Dr. Erica Ollmann Saphire, Kathryn Weinell, The Scripps Research Institute, La Jolla, CA, personal communication).

These studies established that expression of wild type GPC in human cells results in the generation of a significant sGP1 fraction, reflecting either the functional role the protein serves in the infection process (*e.g.*, binding to host cell glycans) or to a weak interaction between GP1 and GP2 on the cell surface that readily leads to dissociation of this complex. In our studies, sGP1 expressed from a wild type GPC molecule consistently yielded largely monomeric protein in the extracellular environment of transfected cells. In contrast, secreted GP1 from sGP1-FLAG consistently resulted in the generation of dimer and trimer species. As analysis of sGP1 protein in these studies was performed under denaturing and reducing conditions, homodimer and homotrimer species may have represented covalently associated subunits. A likely explanation for the presence of significant levels of sGP1 in the supernatants of cells transfected with wild type GPC is glycoprotein ectodomain shedding. This phenomenon has been widely reported and characterized in Ebola virus (EBOV) glycoprotein expression, which has similar features to that of LASV and other arenaviral GPCs [21-25]. Dolnik et al. (2004) reported that the abundant release of EBOV surface glycoprotein GP in a soluble form from virus-infected cells was mediated by cellular shedases. These studies also showed that tumor necrosis factor α-converting enzyme (TACE), a member of the ADAM family of zinc-dependent metalloproteinases, is involved in EBOV GP shedding from virus infected cells. Evidence of significant amounts of shed GP in the blood of EBOV-infected animals was obtained in these studies, and a correlation was established between high levels of the sGP and pathogenesis via efficient blocking of the activity of virus-neutralizing circulating antibodies. Preliminary studies in our laboratory indicate that, unlike with EBOV GP, ectodomain shedding mediated by shedases (zinc-dependent mettaloproteinases) is not a mechanism by which LASV sGP1 is secreted into the extracellular environment of GPC-expressing mammalian cells (unpublished observations). The current proposed model for arenaviral glycoprotein complex structure is comprised of membrane-anchored GP2 homotrimers with non-covalently associated GP1 homotrimers [17]. Thus, it is more likely that secreted GP1 represents an intracellularly cleaved and processed protein fraction that does not associate with GP2 and SP. Nevertheless, the similarity between EBOV and LASV secreted glycoprotein components establishes a possible mechanistic and pathogenic correlate among hemorrhagic fever viruses. It is well established that proteolytic cleavage of GPC processing in arenaviruses is the mechanism by which GP1 and GP2 are generated in virus-infected cells. To date, it has not been shown that arenaviral pre-GPC messenger RNA undergoes alternate splicing to generate a soluble form of GP1. Thus, extracellular arenaviral GP1 is likely to emerge as a result of secretion of this protein throughout the assembly and maturation stages of the membrane-anchored glycoprotein complex during viral biogenesis. The implications for secreted GP1 in viral pathogenesis in humans is not known at this time, but further studies will be aimed at establishing possible correlates, particularly as they relate to early diagnosis of hemorrhagic fevers.

The glycosylation pattern present in sGP1 and sGP2 variants was analyzed by treating purified proteins with the amidases PNGase F and Endo H. Treatment with both amidases resulted in the reduction of the molecular weights of sGP1 and sGP2. These data confirmed that both protein species were glycosylated and that high mannose glycans accounted for this modification. Many glycosylated proteins generated in mammalian cells contain high mannose glycan side groups, and their role in correct protein folding, function and stability have been well documented [26,27]. Although it has been recently reported by Branco et al.,2008 [28] that LASV GP1 and GP2 can be successfully expressed and purified as full length proteins from Escherichia coli (*E. coli*), both lack post-translational modifications, namely glycosylation. The bacterially expressed proteins were readily recognized by mAbs raised against native viral antigen preparations, as well as by antibodies in LASV convalescent human sera. These results should not, however, preclude the expression, purification, and utilization of quasi-native glycoproteins in the development of highly sensitive diagnostic assays. It is likely that antibodies raised against conformational and modified epitopes could represent an important fraction of the Ig component in infected and convalescent patients. Modern mammalian cell line-based expression platforms have significantly reduced the costs associated with generation of recombinant proteins on a volumetric basis. Several highly efficient platforms exist that permit the generation of tens to a few hundred milligrams of recombinant protein per liter of culture in less than one week (Freestyle 293F Expression System, CHO-S and CHO DG44 cells and media kit e.g., Invitrogen). High expression levels of quasi-native proteins coupled to rapid and efficient affinity chromatography-based purification methods make a mammalian expression platform competitive in the generation of important diagnostic proteins with extended functional properties.

## Conclusion

The work reported here provides a basis for developing reagents that could be used in diagnostic platforms and applied to studies aimed at developing countermeasures to thwart Lassa fever. Furthermore, the mechanisms of arenaviral glycoprotein gene expression elucidated in this report establish a means by which to investigate potentially highly relevant aspects of early diagnosis and disease progression in hemorrhagic fevers.

## Methods

### Cell culture, LASV propagation, RNA preparation, cDNA synthesis

HEK-293T/17 cells (ATCC CRL11268) were maintained in complete high glucose Dulbecco's Modified Eagle Medium (cDMEM) supplemented with non-essential amino acids (NEAA) and 10% heat-inactivated fetal bovine serum (ΔFBS).

Vero cells (ATCC CRL 1587) were maintained in complete Eagle's Minimal Essential Medium (cEMEM) supplemented with NEAA, 10% ΔFBS, and 20 μg/mL gentamicin. Vero cells were infected with LASV strain Josiah, [29] at a multiplicity of infection of 0.1. Briefly, virus was diluted in cEMEM to a final volume of 2.0 mL, then added to confluent cells in a T-75 flask and incubated for 1 hour (h) at 37°C, 5% CO_2 _with periodic rocking. Subsequently, 13 mL of cEMEM was added, and the culture was incubated in a similar manner for 96 h. To prepare total cellular RNA, the cell culture medium was replaced with 2 mL of TRIzol reagent (Invitrogen, Carlsbad, CA), and total RNA was purified according to the manufacturer's specifications. Total cellular RNA was reverse transcribed with the ProtoScript First Strand cDNA Synthesis Kit (New England Biolabs, Ipswich, MA), as outlined in the manufacturer's protocol. All LASV gene sequence variants were amplified from cDNA using Phusion™ High-Fidelity polymerase chain reaction (PCR) Mastermix (New England Biolabs).

### Construction of plasmids for protein expression

For LASV protein expression in mammalian cells, pcDNA3.1(+) (Invitrogen) was used as the plasmid vector backbone for all constructs. The vector was modified to contain the entire 1.5-kbp CMV-MIE intron-A (intA) sequence immediately downstream of the parental plasmid's CMV promoter, and was designated pcDNA3.1(+):intA. All LASV gene variants were subcloned as blunt-ended PCR products into the intermediary vector pCR-Blunt II-TOPO^® ^(Invitrogen) and were subsequently cloned via the *Nhe*I and *Hind*III restriction sites within the multiple cloning site of pcDNA3.1(+):intA. The cloning strategy for all variants of LASV GPC, GP1, and GP2 gene sequences into the mammalian expression vector is outlined in Figure 1. DNA was manipulated by standard techniques [30], and all recombinant plasmids were engineered and propagated in *Escherichia coli *(*E. coli*) strain DH5α, according to the manufacturer's instructions (Invitrogen). The DNA sequence and rationale for all oligonucleotides employed in the generation of expression constructs are outlined in Table 1 and in Additional file 1. The accuracy of all LASV GPC, GP1, and GP2 constructs was confirmed by double stranded DNA sequencing (Macrogen, Rockville MD).

**Table 1:** Oligonucleotide primers used for amplification of LASV genes expressed in mammalian cells (see Additional file 1)

**LASV gene amplified**	**LASV primer**	**Oligonucleotide primer sequence**
GPC	5' GPC	GTAGCTAGCATGGGACAAATAGTGACATTCTTCCAG
	3' GPC	GGTACCAAGCTT**TCA**G**TCA**TCTCTTCCATTTCACAGGCAC

GPC-FLAG	5' GPC	
	3' GPC-FLAG	CGATAAGCTT**TCA**G**TCA***GCCCTTGTCGTCGTCGTCCTTGTAGTC*TCTCTTCCATTTCACAGGCAC

sGP1	5' GPC	
	3' sGP1	GGTACCAAGCTT**TCA**G**TCA**TAGCAATCTTCTACTAATATAAATATCTCT
sGP1-FLAG	5' GPC	
	3' sGP1-FLAG	CGATAAGCTT**TCA**G**TCA***GCCCTTGTCGTCGTCGTCCTTGTAGTC*TAGCAATCTTCTACTAATATA

sGP1-hHC	5' hHC-sGP1	GATCGCTAGCGCCGCCACCATG**GGCTGGAGCTGCATCATCCTGTTCCTGGTGGCCACCGCCACCGGCGTGCACAGC**ACCAGTCTTTATAAAGGGGTT
	3' sGP1	

sGP1-hλLC	5' hλLC-sGP1	AAGCTGGCTAGCCACCATG**GCCTGGTCTCCTCTCCTCCTCACTCTCCTCGCTCACTGCACAGGGTCCTGGGCCCAG**ACCAGTCTTTATAAAGGGGTT
	3' sGP1	

GP1-TM	5' GPC	
	3' GP1-TM	GGTACCAAGCTT**TCA**G**TCA***TGGTATTTTGACTAGGTGAAGGAAGATGCTAATAAGATAGAAACTTGTGCTGAACACAAAGAGGTCAACTAGACCCAATGG*TAGCAATCTTCTACTAATATAAATATCTCT

sGP2-GPC SP	5' GPC	
	sGP2-GPC SP	**CAGTGTCCATGTGAATGTGCC***GGTTGTGCAAGACCATCCACACAA*
		
	5' GPC	
		
	3' GP2preTM	GGTACCAAGCTT**TCA**G**CTA**TGTCTTCCCCTGCCTCTCCAT

sGP2-hHC	5' hHC-sGP2	GATCGCTAGCGCCGCCACCATG**GGCTGGAGCTGCATCATCCTGTTCCTGGTGGCCACCGCCACCGGCGTGCACAGC**GGCACATTCACATGGACACTG
		
	3' sGP2preTM	

sGP2-hHC-TM	5' hHC-sGP2	
		
	3' GP2-TM	GGTACCAAGCTT**TCA**G**TCA***TGGTATTTTGACTAGGTGAAGGAA*

sGP2-hHC-FLAG	5' hHC-sGP2	
		
	3' GP2preTM-FLAG	CGATAAGCTT**TCA**G**TCA***GCCCTTGTCGTCGTCGTCCTTGTAGTC*TGTCTTCCCCTGCCTCTCCAT

GP2-hHC-TM-FLAG	5' hHC-sGP2	
		
	3' GP2-TM-FLAG	CGATAAGCTT**TCA**G**TCA***GCCCTTGTCGTCGTCGTCCTTGTAGTC*TGGTATTTTGACTAGGTGAAGGAA

s GP2-hλLC	5' hλLC-sGP2	AAGCTGGCTAGCCACCATG**GCCTGGTCTCCTCTCCTCCTCACTCTCCTCGCTCACTGCACAGGGTCCTGGGCCCAG**GGCACATTCACATGGACACTG
		
	3' sGP2preTM	

sGP2-hλLC-TM	5' hλLC-sGP2	
		
	3' GP2-TM	

GPCΔTMΔIC	5' GPC	
		
	3' GP2preTM	

GPCΔTMΔIC-FLAG	5' GPC	
		
	3' GP2preTM-FLAG	CGATAAGCTT**TCA**G**TCA***GCCCTTGTCGTCGTCGTCCTTGTAGTC*TGTCTTCCCCTGCCTCTCCAT

GPCΔTM-FLAG	5' GP2 TM deletion	AAAATACCAACTCATAGGCATATTGTAG
		
	3' TM deletion	GGAGTATATGGAGAGGCAGGGGAAGACA

GPCΔ_59–249Δ_TMΔIC	5'GPC	
		
	3' SKI-1/GP2	CAGTGTCCATGTGAATGTGCC**TAGCAATCTTCTACTAATATAAATATCTCT***GGTTGTGCAAGACCTACCACACAA*
		
	5' GPC	
		
	3' GP2preTM	

GPCΔ_59–249Δ_TMΔIC-FLAG	5'GPC	
		
	3' SKI-1/GP2	
		
	5' GPC	
		
	3' GP2preTM-FLAG	

GPCΔ_59–249Δ_TM	5' GPC	
		
	3' SKI-1/GP2	
		
	5' GPC	
		
	3' GPC-FLAG	

### Transient expression of LASV gene constructs

Recombinant LASV protein expression was analyzed in HEK-293T/17 cells transiently transfected with mammalian expression vector DNAs, which were prepared using the PureLink HiPure plasmid filter midiprep kit (Invitrogen). The negative control vector pcDNA3.1(+):intA was included in all transfections. Briefly, 1 × 10^6 ^cells were seeded per well of a Poly-D-Lysine-coated 6-well plate in 2 mL of cDMEM. After overnight incubation at 37°C, 5% CO_2_, 90% relative humidity (RH) cells were transfected with unrestricted recombinant plasmid DNAs using the cationic lipid reagent Lipofectamine™ 2000 (Invitrogen), according to the manufacturer's instructions. Four μg of each plasmid DNA were used per transfection. In co-transfection experiments a total of 8 μg were used per reaction (e.g. 4 μg sGP1 and 4 μg sGP2-hHC). Transfections were incubated for 72 h at 37°C, 5% CO_2_, 90% RH after which cell culture supernatants were collected and clarified by centrifugation. To prepare cell extracts from transfected cultures, cell monolayers were carefully washed twice with Ca^++^- and Mg^++^-free PBS, pH7.4, collected by gentle dislodging, transferred to 1.5 mL polypropylene tubes, and lysed for 10 minutes in a mammalian cell lysis buffer comprised of 50 mM Tris buffer, pH 7.5, 1 mM EDTA, 0.1% SDS, 0.5% deoxycholic acid, 1% Igepal CA-360, and a protease inhibitor cocktail (Sigma Aldrich, St. Louis, MO), according to the manufacturer's instructions. The insoluble fraction was pelleted by centrifugation at 14,000 × g for 10 minutes, and the supernatants were transferred to fresh tubes. Protein concentration was determined for each sample with a Bradford assay kit, as indicated by the manufacturer (Pierce, Rockford, IL).

### Western blot analysis of recombinant LASV proteins

Expression of LASV proteins in mammalian cells was confirmed by Western blot analysis using anti-LASV GP1 or GP2 specific mAbs, or M2-FLAG mAb (Sigma Aldrich). Briefly, 10 μg of total cell protein in 10 μL (~1 × 10^5 ^cell equivalents) or 20 μL of cell culture supernatant were resolved by SDS-PAGE in 10% NuPAGE Novex Bis-Tris gels, according to the manufacturer's specifications (Novex, San Diego, CA). All samples in these studies were denatured and reduced in SDS-PAGE buffer containing DTT. Proteins were transferred to 0.45-μm nitrocellulose membranes using XCell II™ Blot Modules, according to the manufacturer's instructions (Invitrogen). Blocking and probing of membranes were performed in 1× PBS, pH 7.4, 5% non-fat dry milk, 0.05% Tween-20, and 0.1% thymerosal. Membranes were washed with 1× PBS, pH 7.4, 0.1% Tween-20 (wash buffer), then probed for 1 hour at room temperature in 10–15 mL of blocking buffer containing 1 μg/mL of relevant murine mAb, washed and incubated for an additional hour with Horseradish peroxidase (HRP)-conjugated goat anti-mouse IgG (H+L) antibody reagent in blocking buffer. Membranes were then washed extensively and developed with TMB membrane substrate purchased from Kirkegaard and Perry Laboratories (KPL, Gaithersburg, MD). Reactions were stopped by immersing developed membranes in water, followed by immediate high resolution scanning for permanent recording.

### PNGase F and Endo H assays

The glycosylation patterns in recombinantly expressed sGP1 and sGP2 were resolved by treatment with the glycosidases PNGase F and Endo H. Twenty μL of FLAG M2 resin-purified proteins from supernatants of HEK-293T/17 cells transiently transfected with sGP1-FLAG, GPCΔTM-FLAG, or GPCΔTMΔIC-FLAG were subjected to treatment with 500 U of PNGase F or Endo H for 1 hour using the reaction conditions suggested by the manufacturer (New England Biolabs). Control reactions were similarly processed except that enzymes were not added. Following incubation proteins were resolved by reducing SDS-PAGE, blotted, probed with anti-FLAG M2 mAb and secondary reagent, and developed as described above.

### Purification of FLAG-tagged sGP1 and sGP2 from culture supernatants

To purify sGP1-FLAG and sGP2-FLAG proteins, 1 mL of supernatant harvested from 293T/17 cells transiently transfected with construct sGP1-FLAG, GPCΔTM-FLAG, or GPCΔTMΔIC-FLAG was clarified by brief centrifugation at 14,000 × *g *and subjected to ANTI-FLAG M2 affinity gel (Sigma Aldrich)-based immunoprecipitation (IP). Briefly, 1 mL of cleared supernatant was added to 40 μL of washed resin and agitated in a roller shaker for 2 hours at room temperature. The gel was then pelleted by brief centrifugation at 6,000 × *g *and the supernatant was transferred to a fresh tube. This supernatant was used to analyze the unbound fraction of FLAG-tagged protein. The gel was washed three times with wash buffer (50 mM Tris HCl, pH 7.4, 150 mM NaCl), followed by resuspension in 100 μL of wash buffer containing 150 ng/mL of 3× FLAG peptide (elution buffer). The samples were incubated with agitation in a roller shaker for 30 minutes at 4°C to elute bound proteins. The gel was subsequently centrifuged at 6,000 × *g *for 30 seconds, and the supernatants containing the eluted protein were transferred to a fresh tube. Twenty μL of each supernatant were subjected to reducing SDS-PAGE and western blot analyses, as described above.

### Monoclonal antibodies to LASV proteins

For immunoassays, Dr. Randy Schoepp (Diagnostic Systems Division, USAMRIID, Ft. Detrick, MD) kindly provided the following LASV-specific monoclonal antibodies (mAbs): GP1-specific mAb L52-74-7A IgG1; GP2-specific mAbs L52-272-7 IgG1 and L52-121-22 IgG2a. These mAbs were raised against purified gamma-irradiated LASV, as previously described [31].

## Competing interests

Megan M Illick is a scientist at BioFactura, Inc. and has received salary and other compensation from the company, such as incentive stock options, as it pertains to the execution of this work. Luis M Branco served as Chief Scientific Officer and a member of the Board of Directors of BioFactura, Inc., until September 5, 2008, and has received salary and other compensation from the company, such as founder's stock options, as it pertains to the execution of this work. Alex Matschiner is a co-founder and chairman of the Board of Directors of BioFactura, Inc., and has received salary and other compensation from the company, such as founder's and incentive stock options, as it pertains to the execution of this work. This publication may, in part, result in the seeking of additional funding, public or private, to support follow-up studies pertinent to the work outlined herein. Luis M Branco, Megan M Illick, Alex Matschiner, Joseph N Fair, Robert F Garry, and Mary C Guttieri are listed inventors, in addition to others, in a PCT application entitled "Soluble and Membrane-Anchored Forms of Lassa Virus Subunit Proteins", filed in April 2008.

## Authors' contributions

MMI engineered the expression systems and contributed to the drafting of the manuscript. LMB contributed to the experimental design, engineered the expression systems, performed data analysis, drafted, and edited the manuscript. JNF contributed to the cloning of LASV genes and procurement of critical reagents. KAI contributed to the engineering of expression systems and *in vitro *expression experiments. AM developed purification methods for each of the proteins. RJS provided LASV-specific mAbs. RFG contributed to the experimental design and provided critical review of the manuscript. MCG contributed to the experimental design, procurement of critical reagents, data analysis, drafting and critical review of the manuscript.

**Figure 7:**
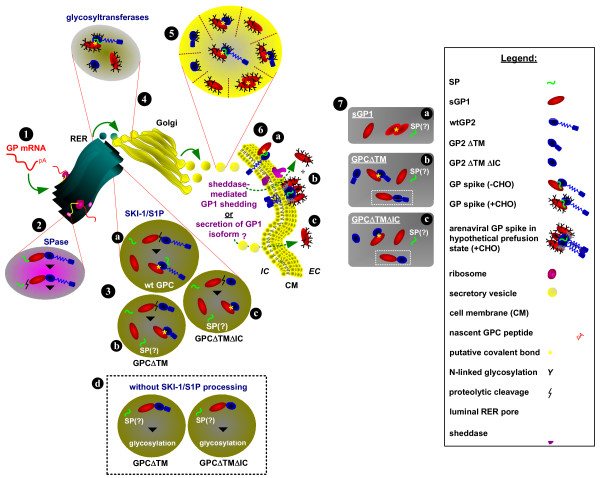
**Hypothetical arenaviral glycoprotein expression pathway and secretory forms. **Arenaviral glycoprotein (GP) mRNA is transcribed from plasmid DNA constructs in the host cell (1), and directed to the rough endoplasmic reticulum (RER) for translation. The nascent polyprotein is directed to the RER lumen by the SP, where SPases cleave SP from the GP polyprotein precursor (2). In the RER the GP precursor is cleaved by SKI-1/S1P to yield GP1 and GP2 (3). Wild-type GP may be comprised of covalently-linked GP1 and GP2, and associated SP (3a). In GPCΔTM (3b) and GPCΔTMΔIC (3c) truncated GP2 subunits may associate covalently with GP1. The role of SP in these variants is unknown. A GP fraction expressed from constructs GPCΔTM and GPCΔTMΔIC that is not processed by SKI-1/S1P could shuttle uncleaved polyprotein to the glycosylation pathway (3d). The SP’s role here is also unknown. Glycosylation of GP1 and GP2 is mediated by RER or Golgi glycosyltransferases (4). Membrane-bound vesicles transport proteins to the Golgi *cis* face where processing and assembly occur. Assembled and glycosylated GP complex or subunits are packaged in exocytic vesicles that emerge from the Golgi *trans* face and are directed toward the plasma membrane (5). The GP complex is anchored via the GP2 transmembrane domain (6a, b). Complexed heterodimeric GP1–GP2 is displayed in 6a, whereas GP spike trimer in a hypothetical prefusion form is shown in 6b. Extracellular GP1 from expression of wild-type GPC may be produced by protease-mediated ectodomain shedding, or an isoform of secreted GP1. The potential forms of the GP complex and subunits are outlined in 7. Monomeric sGP1 and homodimer (7a), soluble monomeric GP2 and GP1, complexed heterodimer, or the potentially uncleaved GP precursor (in etched box) derived from GPCΔTM (7b) and GPCΔTMΔIC (7c) are shown. Legend box: Graphic representations of relevant components**.**

## Supplementary Material

Additional file 1 Detailed oligonucleotide primers and methods used for amplification of LASV genes expressed in mammalian cells. Detailed oligonucleotide sequences, outline of functional expression elements, and PCR methods employed in these studies. Click here for file
