# Supplementary material for: Uncoupling GP1 and GP2 expression in the Lassa virus glycoprotein complex: implications for GP1 ectodomain shedding
Source: Virol J. 2008 Dec 23;5:161. doi: 10.1186/1743-422X-5-161 (PMC2645378; doi:10.1186/1743-422X-5-161)
Supplement: Additional file 1 — Detailed oligonucleotide primers and methods used for amplification of LASV genes expressed in mammalian cells. Detailed oligonucleotide sequences, outline of functional expression elements, and PCR methods employed in these studies. [file 1743-422X-5-161-S1.doc]

The LASV GPC ORF, which encodes the native SP through the C-terminus of the GP2 IC domain (amino acids [a.a.]: 1-491) was amplified with primers 5’ GPC (GTAGCTAGCATGGGACAAATAGTGACATTCTTCCAG; the *NheI* site is underlined, the start codon [ATG] is double-underlined) and 3’ GPC (GGTACCAAGCTT**TCA**G**TCA**TCTCTTCCATTTCACAGGCAC; the *HindIII* site is underlined, the stop codons [TCA] are in boldface type). Similarly, GPC was designed with the FLAG-tag sequence (DYKDDDDKG) fused to the C-terminus of GP2 at a.a. 491 to facilitate detection and purification with anti-FLAG resin. GPC-FLAG was amplified with primers 5’ GPC and 3’ GPC-FLAG (CGATAAGCTT**TCA**G**TCA***GCCCTTGTCGTCGTCGTCCTTGTAGTC*TCTCTTCCATTTCACAGGCAC; the *HindIII* site is underlined, the stop codons [TCA] are in boldface type, the FLAG sequence is italicized). The pcDNA3.1(+):intA:GPC and pcDNA3.1(+):intA:GPC-FLAG plasmids were subsequently used for generation of sGP1 and sGP2 variants.

The sGP1 gene containing the native SP though the C-terminal end of mature GP1 (a.a. 1-259) was amplified from GPC with primers 5’ GPC and 3’ sGP1 (GGTACCAAGCTT**TCA**G**TCA**TAGCAATCTTCTACTAATATAAATATCTCT; the *HindIII* site is underlined, the stop codons [TCA] are in boldface type). Soluble

GP1-FLAG was amplified from GPC with primers 5’ GPC and 3’ sGP1-FLAG (CGATAAGCTT**TCA**G**TCA***GCCCTTGTCGTCGTCGTCCTTGTAGTC*TAGCAATCTTCTACTAATATA; the *HindIII* site is underlined, the stop codons [TCA] are in boldface type, the FLAG sequence is italicized) by fusing the FLAG tag sequence to the C-terminus of mature GP1 at a.a. 259. Two additional sGP1 variants were generated that differed only in the SP sequence: sGP1 containing a human HC signal sequence fused to the N-terminus of GP1 at a.a. 59, designated sGP1-hHC, was amplified with primers 5’ hHC-sGP1 (GATCGCTAGCGCCGCCACCATG**GGCTGGAGCTGCATCATCCTGTTCCTGGTGGCCACCGCCACCGGCGTGCACAGC**ACCAGTCTTTATAAAGGGGTT; the *NheI* site is underlined, the start codon [ATG] is double-underlined, the hHC signal sequence is in boldface type) and 3’ sGP1. Similarly, sGP1 containing a human λLC signal sequence fused to the N-terminus of GP1 at a.a. 59, designated sGP1-h λLC, was amplified with primers 5’ hλLC-sGP1 (AAGCTGGCTAGCCACCATG**GCCTGGTCTCCTCTCCTCCTCACTCTCCTCGCTCACTGCACAGGGTCCTGGGCCCAG**ACCAGTCTTTATAAAGGGGTT; the *NheI* site is underlined, the start codon [ATG] is double-underlined, the hλLC signal sequence is in boldface type) and 3’ sGP1. A membrane-anchored GP1 variant that contained the native GPC SP and the TM domain of mature GP2 (a.a. 427-453) fused to the C-terminal ectodomain of GP1 at a.a. 259, and designated GP1-TM, was amplified with primers 5’ GPC and 3’ GP1-TM (GGTACCAAGCTT**TCA**G**TCA***TGGTATTTTGACTAGGTGAAGGAAGATGCTAATAAGATAGAAACTTGTGCTGAACACAAAGAGGTCAACTAGACCCAATGG*TAGCAATCTTCTACTAATATAAATATCTCT; the *HindIII* site is underlined, the stop codons [TCA] are in boldface type, the TM domain of mature GP2 is italicized, the C-terminal 10a.a. of GP1 is double underlined).

The ectodomain of LASV GP2, beyond the pre-GPC cleavage site catalyzed by the SKI-1/S1P protease (RRLL↓) and ending just prior to the TM domain (a.a 260-427) was cloned for expression as a soluble protein. The same three signal sequences used for expression of sGP1 were fused to the N-terminus of sGP2 constructs. The native GPC SP contained a native translation initiation motif, and it was fused to the first amino acid of mature GP2 (Gly). The human Ig signal sequences (hHC or hλLC) contained an optimal Kozak sequence (CCACCATGG), including an N-terminal Met. The C-terminus of GP2 constructs was engineered to contain two termination codons interspersed by a single nucleotide. The FLAG-tag in GP2-FLAG comprised the terminal 9 amino acids prior to the termination codon. The construct sGP2-GPC SP was generated by PCR-mediated ligation. Briefly, an antisense sGP2 GPC SP primer was synthesized that contained the 3’ terminal 24 nucleotides of the GPC SP fused to the N-terminal 21 nucleotides of mature GP2 (**CAGTGTCCATGTGAATGTGCC***GGTTGTGCAAGACCATCCACACAA*; the GPC SP sequence is italicized, the N-terminal 21 nucleotides of GP2 are in boldface type). This primer was used in a PCR reaction with sGP1 construct and primer 5’ GPC to amplify a 204 bp cassette. The PCR product was diluted 1:100 and used in a second PCR reaction containing sGP2-hHC construct, and amplified with primers 5’ GPC and 3’ GP2preTM (GGTACCAAGCTT**TCA**G**CTA**TGTCTTCCCCTGCCTCTCCAT; the *HindIII* site is underlined, the stop codons [TCA and CTA] are in boldface type). For generation of sGP2-hHC or sGP2-hλLC the N-terminus of the mature GP2 sequence (a.a. 260-427) was fused to the C-terminus of either the hHC or hλLC signal sequences. sGP2-hHC was amplified with primers 5’ hHC-sGP2 (GATCGCTAGCGCCGCCACCATG**GGCTGGAGCTGCATCATCCTGTTCCTGGTGGCCACCGCCACCGGCGTGCACAGC**GGCACATTCACATGGACACTG; the *NheI* site is underlined, the start codon [ATG] is double-underlined, the hHC sequence is in boldface type) and 3’ sGP2preTM. Soluble GP2-hHC was further modified to contain either the TM domain, a FLAG-tag, or both. Soluble GP2-hHC with the TM domain, designated sGP2-hHC-TM, was amplified with primers 5’ hHC-sGP2 and 3’ GP2-TM (GGTACCAAGCTT**TCA**G**TCA***TGGTATTTTGACTAGGTGAAGGAA*; *Hind*III site is underlined, the stop codons [TCA] are in boldface type, TM domain is italicized). Soluble GP2-hHC with a FLAG-tag, designated sGP2-hHC-FLAG, was amplified with primers 5’ hHC-sGP2 and 3’ GP2preTM-FLAG (CGATAAGCTT**TCA**G**TCA***GCCCTTGTCGTCGTCGTCCTTGTAGTC*TGTCTTCCCCTGCCTCTCCAT; *Hind*III site is underlined, the stop codons [TCA] are in boldface type, the FLAG sequence is italicized, GP2 preTM sequence is double underlined). The sGP2-hHC variant containing both the TM domain and FLAG-tag, designated GP2-hHC-TM-FLAG, was amplified with primers 5’ hHC-sGP2 and 3’ GP2-TM-FLAG (CGATAAGCTT**TCA**G**TCA***GCCCTTGTCGTCGTCGTCCTTGTAGTC*TGGTATTTTGACTAGGTGAAGGAA; *Hind*III site is underlined, the stop codons [TCA] are in boldface type, the FLAG sequence is italicized, GP2-TM sequence is double underlined). Soluble GP2-hλLC was amplified with primers 5’ hλLC-sGP2 (AAGCTGGCTAGCCACCATG**GCCTGGTCTCCTCTCCTCCTCACTCTCCTCGCTCACTGCACAGGGTCCTGGGCCCAG**GGCACATTCACATGGACACTG; the *NheI* site is underlined, the start codon [ATG] is double-underlined, the hλLC sequence is in boldface type) and 3’ GP2preTM. Soluble GP2-hλLC was further modified to contain the TM domain of mature GP2, and designated sGP2-hλLC-TM, which was amplified with primers 5’ hλLC-sGP2 and 3’ GP2-TM.

GPCΔTMΔIC encodes the GPC SP through the C-terminal a.a. of GP2 prior to the TM domain (a.a. 1-426) and was amplified with primers 5’ GPC and 3’ GP2preTM. GPCΔTMΔIC-FLAG is identical to GPCΔTMΔIC, except that a FLAG tag was added to the C-terminus of GP2 prior to the TM domain at a.a. 426. GPCΔTMΔIC-FLAG was amplified with primers 5’ GPC and 3’ GP2preTM-FLAG (CGATAAGCTT**TCA**G**TCA***GCCCTTGTCGTCGTCGTCCTTGTAGTC*TGTCTTCCCCTGCCTCTCCAT; the *HindIII* site is underlined, the stop codons (TCA) are in boldface type, the FLAG sequence is italicized).

GPCΔTM-FLAG encodes the entire GPC-FLAG minus the TM domain of GP2 with the remaining sequence including the SP (a.a. 1-58), GP1 (a.a. 59-259), GP2 prior to the TM domain (a.a. 260-426), and the IC domain of GP2 (a.a. 451-491). The GPCΔTM-FLAG was amplified from the template pcDNA3.1(+):intA:GPC-FLAG with primers 5’ GP2 TM deletion (AAAATACCAACTCATAGGCATATTGTAG) which includes 9 a.a. at the N-terminal end of the GP2 IC domain, and 3’ TM deletion (GGAGTATATGGAGAGGCAGGGGAAGACA) which includes 9 a.a. from the C-terminus of GP2 prior to the TM domain. The resulting PCR product was treated with bacteriophage T4 polynucleotide kinase (T4 PNK), and religated to generate a construct containing a ΔTM variant.

Constructs GPCΔTMΔIC, GPCΔTMΔIC-FLAG, and GPCΔTM-FLAG were further modified by removing a.a 59-249 of the GP1 domain and designated GPCΔ_59-249_ΔTMΔIC, GPCΔ_59-249_ΔTMΔIC-FLAG, and GPCΔ_59-249_ΔTM-FLAG, respectively. The remaining GP1 domain contains only the C-terminal 10 a.a. (RDIYISRRLL) of mature GP1 spanning the SKI-1/S1P cleavage junction (RRLL↓). The GPCΔ_59-249_ΔTMΔIC construct, which encodes the native SP, ten C-terminal a.a. of GP1 including the SKI-1/S1P site, and GP2 up to the TM domain was generated by PCR-mediated ligation. Using the GPC construct as a template, the first PCR reaction amplified the SP, 10 a.a. of GP1, and 7 N-terminal a.a. of GP2 (GTFTWTL) using primers 5’GPC and 3’ SKI-1/GP2 (CAGTGTCCATGTGAATGTGCC**TAGCAATCTTCTACTAATATAAATATCTCT***GGTTGTGCAAGACCTACCACACAA*; the GP2 sequence is underlined, the C-terminal GP1 sequence is in boldface type, the SP sequence is italicized). The PCR product generated from the 5’GPC and 3’ SKI-1/GP2 reaction was diluted 1:100 and used in a second PCR reaction containing pcDNA3.1(+):intA:GPC with primers 5’ GPC and 3’ GP2preTM to generate GPCΔ_59-249_ΔTMΔIC. GPCΔ_59-249_ΔTMΔIC:FLAG, which is identical to GPCΔ_59-249_ΔTMΔIC but includes the addition of a FLAG-tag, and was produced in the same manner except that the PCR product generated from amplification with 5’GPC and 3’ SKI-1/GP2 was used in the second reaction with pcDNA3.1(+):intA:GPC and primers 5’ GPC and 3’ GP2preTM-FLAG. GPCΔ_59-249_ΔTM was generated by combining the PCR product template amplified from 5’ GPC and 3’ SKI-1/GP2 and the TM domain lacking GPCΔTM-FLAG with primers 5’ GPC and 3’ GPC-FLAG.
